# Development of the Pelvic Reduction Grading System (PRGS): A Radiographic Tool for Assessing Alignment After Pelvic Ring Fixation

**DOI:** 10.51894/001c.164037

**Published:** 2026-07-31

**Authors:** Saif L. Juma, Kamil R. Jarjess, Chayton Fivecoat, Sasha Stine, Martina Stojanovska, Rahul Vaidya

**Affiliations:** 1 College of Osteopathic Medicine, Michigan State University

## 22

### BACKGROUND

Pelvic ring injuries are complex fractures requiring accurate reduction and stable fixation to restore pelvic symmetry and optimize functional outcomes. Although several radiographic techniques assess pelvic alignment, a standardized grading system for evaluating postoperative reduction using a single radiographic view has not been established.

### OBJECTIVES

To introduce the Pelvic Reduction Grading System (PRGS), a radiographic grading metric designed to evaluate postoperative alignment following operative fixation of pelvic ring injuries. We hypothesized that the PRGS would distinguish anatomically reduced pelvic fractures from malunited cases using a single postoperative anteroposterior (AP) pelvic radiograph.

### METHODS

A retrospective cohort study was performed at a Level I trauma center. Adult patients who underwent operative fixation of pelvic ring injuries between 2018 and 2024 were identified from an institutional orthopedic trauma database. Patients were included if a postoperative AP pelvic radiograph obtained within 24 hours of surgery was available for analysis. A comparison cohort of patients with established pelvic malunions was also evaluated. The PRGS incorporates three radiographic parameters measured on AP pelvis radiographs: pubic symphysis width normalized to femoral head diameter, pelvic symmetry using the Keshishyan Deformity Index, and vertical hemipelvis displacement. Each parameter meeting predefined thresholds was assigned one point, producing grades from A (3/3 criteria met) to D (0/3 criteria met). Statistical comparisons were performed using chi-square and Mann-Whitney U tests.

### RESULTS

A total of 107 patients were evaluated, including 97 surgically treated pelvic ring injuries and 10 pelvic malunions. In the operative cohort, PRGS grades were distributed as follows: Grade A in 54 patients (55.7%), Grade B in 41 (42.3%), and Grade C in 2 (2.1%). No patients were classified as Grade D. In the malunion cohort, 3 cases were graded as B and 7 as C. PRGS grade distribution differed significantly between cohorts (*p* < 0.001). Median PRGS scores were also significantly higher in the operative cohort (*p* < 0.001).

### CONCLUSION

The PRGS is a simple and reproducible radiographic grading system for evaluating postoperative pelvic alignment using a single AP pelvic radiograph. It may serve as a practical tool for postoperative assessment following pelvic ring fixation.

